# Longitudinal Tracking of Immune Responses in COVID-19 Convalescents Reveals Absence of Neutralization Activity Against Omicron and Staggered Impairment to Other SARS-CoV-2 Variants of Concern

**DOI:** 10.3389/fimmu.2022.863039

**Published:** 2022-03-14

**Authors:** Ivan Odak, Christian R. Schultze-Florey, Swantje I. Hammerschmidt, Christiane Ritter, Stefanie Willenzon, Michaela Friedrichsen, Inga Ravens, Ruth Sikora, Lâle M. Bayir, Rodrigo Gutierrez Jauregui, Günter Bernhardt, Metodi V. Stankov, Anne Cossmann, Guido Hansen, Thomas Krey, Markus Cornberg, Christian Koenecke, Georg M. N. Behrens, Berislav Bošnjak, Reinhold Förster

**Affiliations:** ^1^ Institute of Immunology, Hannover Medical School, Hannover, Germany; ^2^ Department of Hematology, Hemostasis, Oncology and Stem Cell Transplantation, Hannover Medical School, Hannover, Germany; ^3^ Clinic Department for Rheumatology and Immunology, Hannover Medical School, Hannover, Germany; ^4^ Institute of Biochemistry, University of Lübeck, Lübeck, Germany; ^5^ German Center for Infection Research (DZIF), Partner Sites Hamburg-Lübeck-Borstel-Riems, Brunswick, Germany; ^6^ Cluster of Excellence RESIST (EXC 2155), Hannover Medical School, Hannover, Germany; ^7^ German Center for Infection Research (DZIF), Partner Sites Hannover-Braunschweig, Brunswick, Germany; ^8^ Centre for Individualised Infection Medicine (CiiM), Hannover, Germany; ^9^ Department of Gastroenterology, Hepatology and Endocrinology, Hannover Medical School, Hannover, Germany

**Keywords:** antibodies, spike, B cells, protection, variants of concern, omicron, delta

## Abstract

Evaluating long-term protection against SARS-CoV-2 variants of concern in convalescing individuals is of high clinical relevance. In this prospective study of a cohort of 46 SARS-CoV-2 patients infected with the Wuhan strain of SARS-CoV-2 we longitudinally analyzed changes in humoral and cellular immunity upon early and late convalescence. Antibody neutralization capacity was measured by surrogate virus neutralization test and cellular responses were investigated with 31-colour spectral flow cytometry. Spike-specific, isotype-switched B cells developed already during the disease phase, showed a memory phenotype and did not decrease in numbers even during late convalescence. Otherwise, no long-lasting perturbations of the immune compartment following COVID-19 clearance were observed. During convalescence anti-Spike (S1) IgG antibodies strongly decreased in all patients. We detected neutralizing antibodies against the Wuhan strain as well as the Alpha and Delta but not against the Beta, Gamma or Omicron variants for up to 7 months post COVID-19. Furthermore, correlation analysis revealed a strong association between sera anti-S1 IgG titers and their neutralization capacity against the Wuhan strain as well as Alpha and Delta. Overall, our data suggest that even 7 month after the clearance of COVID-19 many patients possess a protective layer of immunity, indicated by the persistence of Spike-specific memory B cells and by the presence of neutralizing antibodies against the Alpha and Delta variants. However, lack of neutralizing antibodies against the Beta, Gamma and Omicron variants even during the peak response is of major concern as this indicates viral evasion of the humoral immune response.

## Introduction

Since the emergence of the new coronavirus SARS-CoV-2 in December 2019, a growing body of literature has been published elucidating the immune responses in COVID-19 patients (1–3). The longevity of the immunological memory post SARS-CoV-2 infection is a matter of public health concern. Recent data on lasting SARS-CoV-2 immunity indicates that antibody levels start to significantly decline around 4 months after the disease onset (4) implying waning of immune protection.

Due to the emergence of novel variants of concern (VoCs) and variants being monitored (VBM), cross-variant immunity is of high public relevance. Currently, the Centers for Disease Control and Prevention (CDC) lists 10 VBM (5). Hence, possible viral escape through mutation has become a subject of increasing interest. This concern grew with recent evidence of antigen divergence of the B.1.351 (Beta) and the P.1 (Gamma, formerly named B.1.28.1) VoC (2, 6). Moreover, the newly discovered B.1.1.529 VoC termed Omicron (7), contains so far the highest number of mutations in the receptor binding domain (RBD), providing fuel to the fear of viral escape of acquired immunity (8, 9). It is likely that the current range of mutation of SARS-CoV-2 is underestimated and further VoCs are already on the rise. In this context, assessing the level of protection conferred by convalescence to (re)infection by VoCs is of great importance.

In the present prospective study of a cohort of 46 SARS-CoV-2 patients infected with the Wuhan strain of SARS-CoV-2 we longitudinally analyze the changes in humoral and cellular immunity upon early and late convalescence. We also provide insights into the range and amplitude of cross-mutational responses against the Alpha (B.1.1.7), Beta, (B.1.351), Gamma (P.1; formerly named B.1.28.1), Delta (B.1.617.2) and Omicron (B.1.1.529) variants, as well as the original Wuhan strain. Our data imply diverging rates of affinity maturation as well as decay of neutralizing antibodies against SARS-CoV-2 VoCs in COVID-19 patients and convalescent individuals, while Spike-specific B cells are shown to persist in circulation for up to seven month post clearance of COVID-19– the maximum observation period of the present study. Most worryingly, regardless of the disease kinetics and severity, all 120 sera sample tested revealed complete absence of neutralizing antibodies directed against the Omicron variant.

## Material and Methods

### Study Participants

A total of 50 hospitalized and ambulatory patients with PCR-confirmed SARS-CoV-2 infection were recruited at Hannover Medical School from March 26^th^ until July 31^st^ 2020. During that period, the Wuhan strain was the dominant variant of SARS-CoV-2 in Germany. Our cohort of 46 patients was predominantly male (65%) and 50% of all patients had pre-existing co-morbidities. Patients’ characteristics are listed in **Table 1**. The median follow-up post onset of symptoms was 151 days (range 3 - 240 days) post onset of symptoms. The study was approved by the institutional review board at Hannover Medical School (#9001_BO_K2020) and informed consent was obtained from all patients. All further cohort details are described in **Supplementary Material and Methods**.

**Table 1:** Patients’ characteristics.

ID	Max WHO Scale	Age	Sex	PEMC*	Symptoms at disease onset	Number of samples	Last sampling^#^
003	2	34	M	no	dry cough, dyspnea, malaise	2	194
004	2	31	F	yes	dyspnea, malaise	2	203
008	2	64	M	yes	fever, dry cough, dyspnea, malaise, anosmia, chest pain	2	232
010	2	58	F	no	dry cough, dyspnea, malaise, anosmia, chest pain	3	184
017	2	61	M	yes	fever, dry cough	1	101
018	2	50	F	no	fever, dyspnea, anosmia, chest pain	2	111
020	2	23	F	no	fever, dyspnea, malaise, anosmia	2	205
037	2	48	M	no	fever, dry cough, malaise, anosmia, chest pain	1	70
047	2	42	M	no	fever, dry cough, dyspnea, anosmia, chest pain, diarrhea	2	216
048	2	57	F	yes	dry cough, malaise, chest pain	1	95
013	3	49	F	yes	fever, dry cough, dyspnea, malaise, chest pain	1	75
021	3	68	M	yes	fever, dry cough, dyspnea, chest pain	3	231
022	3	80	M	yes	fever, malaise	1	18
026	3	48	M	no	dry cough, dyspnea, chest pain	2	179
028	3	48	M	yes	fever, dry cough	1	3
033	3	57	F	no	dry cough, dyspnea, diarrhea	3	215
036	3	23	F	no	fever, dry cough	2	182
043	3	31	F	no	dyspnea, malaise, chest pain	1	63
005	4	69	M	yes	fever, dry cough, dyspnea, malaise, anosmia	2	69
007	4	71	M	no	fever, malaise	2	205
014	4	83	F	yes	fever, dyspnea	4	62
019	4	42	M	yes	dyspnea	3	101
025	4	30	M	no	fever, dry cough, malaise, diarrhea	2	16
011	5	48	F	yes	fever, dry cough, dyspnea, malaise, chest pain	3	208
042	5	63	M	yes	dyspnea, malaise, anosmia, diarrhea	5	137
045	5	48	M	no	fever, dry cough, dyspnea, malaise, anosmia, chest pain, diarrhea	3	205
046	5	18	M	no	fever, dry cough, dyspnea, anosmia	4	122
009	6	68	M	no	fever, diarrhea	2	146
012	6	79	M	yes	dry cough, dyspnea, malaise, diarrhea	3	182
015	6	58	F	yes	fever, dry cough, dyspnea, malaise, chest pain	3	224
016	6	53	M	yes	fever, dry cough, diarrhea	3	209
023	6	75	M	yes	fever, dry cough	2	16
024	6	52	M	yes	fever, malaise, diarrhea	2	218
029	6	62	M	yes	fever, dry cough, dyspnea, malaise, anosmia	4	181
030	6	60	M	no	fever, dyspnea, malaise	3	195
032	6	47	M	no	fever, dry cough, malaise	2	225
034	6	39	M	no	fever, dyspnea	5	240
039	6	67	M	yes	fever, dry cough	2	15
040	6	68	F	yes	dry cough, dyspnea	2	22
041	6	34	F	no	fever, dry cough, dyspnea, malaise, chest pain	5	128
044	6	53	F	no	dyspnea, malaise, anosmia	3	111
050	6	32	M	no	fever, dry cough, diarrhea	3	26
002	7	19	M	yes	fever, dry cough, dyspnea, malaise	5	206
027	7	36	F	no	fever, dry cough, dyspnea, malaise	5	156
038	7	59	M	yes	dry cough, dyspnea, malaise	3	32
049	7	42	M	no	dyspnea	3	32

PEMC, pre-existing medical conditions; m, male; f, female.*cardiovascular, respiratory and/or metabolic PEMC; ^#^days from first symptoms.

### ELISA

Anti SARS-CoV-2 S1 Spike protein IgG antibodies were determined by quantitative ELISA (QuantiVac, Euroimmun) according to the manufacturer’s instructions (dilution 1:400, 1:600, or 1:1000). Antibody levels were calculated as RU/ml from calibration curves, with values above 11 RU/ml defined as positive. All values were then transformed to BAU/ml by multiplying RU values with 3.2 as per assay instructions. Anti–SARS-CoV-2 nucleocapsid protein (NCP) and anti-Sars-CoV2 S1 Spike protein domain immunoglobulin A (IgA) (Euroimmun) was done according to the manufacturer’s instructions. Antibody amounts are expressed as IgA ratio (optical density divided by calibrator). An AESKU.READER (AESKU.GROUP) and the with Gen5 version 2.01 software were used for analysis.

### Surrogate Virus Neutralization Test (sVNT) for SARS-CoV-2 Variants

The sera from convalescent patients were analyzed using sVNT, an ELISA-based assay that determines titers of virus neutralizing antibodies by measuring how potently serum inhibits binding of the RBD of SARS-CoV-2 to surface-immobilized ACE2, as described earlier (10, 11). Previous results from our laboratory revealed high correlation of sVNT results to data obtained in pseudotyped virus neutralization tests for the original Wuhan strain and the Alpha, Beta, Gamma, Delta and Omicron VoCs (10–12).

### Flow Cytometry

Peripheral Blood Mononuclear Cells (PBMCs) were prepared from anticoagulated whole blood as described in detail elsewhere (13). After thawing, cells were stained for 30 min on RT with an antibody mix containing antibodies listed in **Supplementary Table 2** together with a SARS-CoV-2 Spike-mNeonGreen fusion protein (11) (5 μg per reaction). After one wash, samples were acquired on an Aurora spectral flow cytometer (Cytek) equipped with five lasers operating at 355 nm, 405 nm, 488 nm, 561 nm and 640 nm. All flow cytometry data were acquired using SpectroFlo version 2.2.0 (Cytek) and analyzed with FCS Express 7 (Denovo).

### Statistical Analysis

Data were analyzed using Prism 8.4.3 (GraphPad). All data was tested for normality of distribution. For the analysis of unpaired groups, Kruskal-Wallis multiple comparison test with Dunn’s post hoc correction was used, and in case of paired values, two-tailed Wilcoxon matched-pairs signed rank test was used. In both cases, p values p<0.05 were considered significant. For determining correlation, nonparametric Spearman test was used. The statistical analysis applied is indicated in the figure legends.

## Results

### Leukocyte Responses During Convalescence

**Figure 1:**
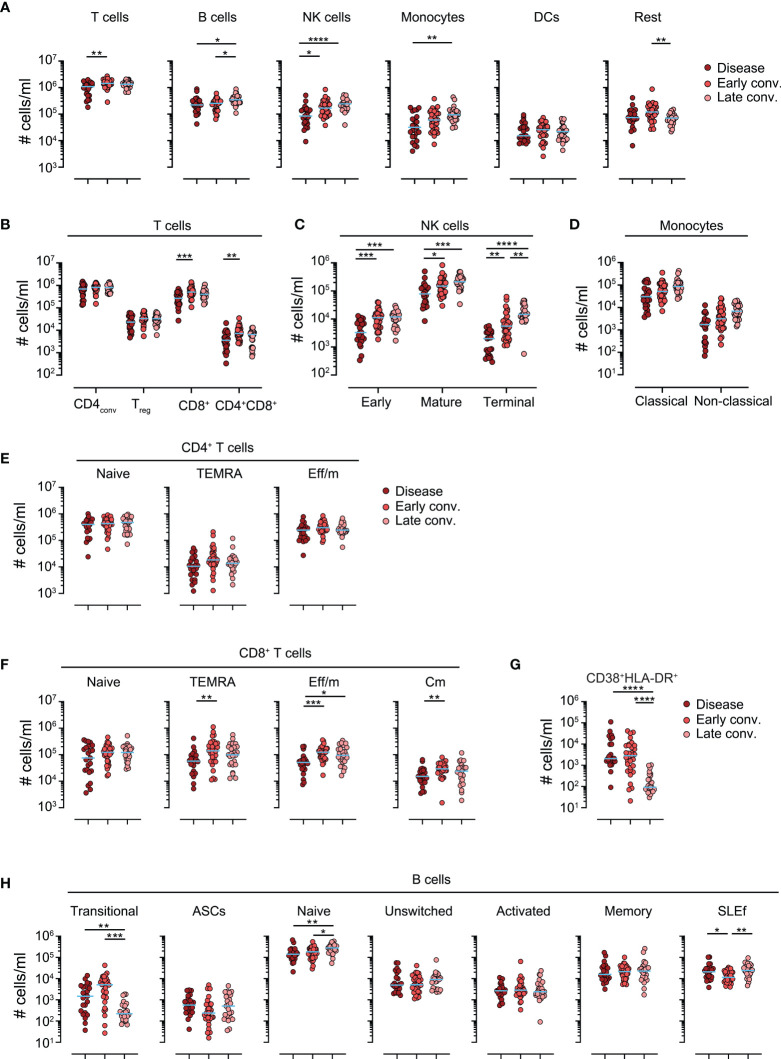
Leukocyte responses in convalescing patients. Absolute cells numbers per ml of blood in patients with disease, early and late convalescence. **(A)** Cell numbers of various leukocyte populations. **(B)** Cell numbers of blood of major T cell subsets **(C)** Cell numbers of blood of NK cell subsets **(D)** Cell numbers of blood of monocyte subsets. **(E)** Absolute cell numbers of memory subsets of conventional CD4^+^ T cells. **(F)** Absolute cell numbers of memory subsets of conventional CD8^+^ T cells. **(G)** Absolute cell numbers of blood of CD8^+^CD38^+^HLA-DR^+^ effector memory subpopulation. **(H)** Absolute cell numbers of B cell subsets. Each dot represents one biologically individual sample taken from one patient. Lines represent median. *N* (disease)=24; *n* (early conv.)=32; *n* (late conv.)=25. Statistics: Kruskal-Wallis multiple comparison test with Dunn’s *post hoc* correction.*p<0.05; **p<0.01; ***p<0.001; ****p<0.0001.

To characterize the involvement and long-term perturbations of immune responses during convalescence we performed an in-depth analysis using spectral flow cytometry. We developed a 31 color staining panel to identify all major leukocyte subsets (**Supplementary Table 2**). The gating principle is shown in **Supplementary Figure 1**. Of all major leukocyte subset tested, we found B cells, and to a greater extent, natural killer (NK) cells and monocytes, to be elevated during convalescent phases. In contrast, T cells and the heterogeneous pool of innate lymphoid cells (ILCs), basophils and other cells (not fulfilling the criteria of any of the listed populations, altogether shown as “Rest”) only displayed a mild transient increase in early convalescence (**Figure 1A** and **Supplementary Figure 2A**). A more detailed analysis of T cells revealed that CD8^+^ and CD4^+^CD8^+^ were the major T cell subsets increasing in early convalescence (**Figure 1B**). NK cells can be subdivided based on their CD56 and CD16 expression in “early” (CD56^bright^CD16^-^), “mature” (CD56^dim^CD16^+^) and “terminal” (CD56^-^CD16^+^) (14, 15) NK cells. Interestingly, we found all three subsets to be significantly elevated in the blood of both early and late convalescing patients compared to disease patients (**Figure 1C**), likely due to general lymphopenia of severe COVID-19 patients (16). However, only terminal NK cells were also increased in frequencies (**Supplementary Figure 2C**). Of note, we observed no differences in numbers or frequencies of monocyte subsets during convalescence (**Figure 1D** and **Supplementary Figure 2D**).

Next, we assessed the memory phenotype of T and B cells. Several major T cell memory subsets, identified by differential CCR7 and CD45RA expression (17), have been shown to play a role in clearing infections. Interestingly, in our cohort, we failed to observe differences in cell numbers or in frequencies of the 3 major CD4^+^ subsets – naïve, TEMRA, effector/memory - between disease and convalescent phases (**Figure 1E**). On the other hand, compared to active disease, early convalescing patients had noticeably expansions of all antigen experienced - TEMRA, effector/memory, central memory - CD8^+^ T cell populations (**Figure 1F**). A comparable trend was followed in cell frequencies, however without reaching statistical significance (**Supplementary Figure 2G**). Importantly, CD38^+^HLA-DR^+^ CD8^+^ T cells have been demonstrated to contain the pool of cells recognizing SARS-CoV-2 antigens (18, 19). We found that pool of cells to be drastically reduced at late convalescence (**Figure 1G** and **Supplementary Figure 2G**) indicating contraction of the T cell response following infection clearance.

Analysis of B cell subpopulations revealed a decrease in transitional CD24^+++^IgM^+++^ B cells during late convalescence, as well as an increase in naïve IgD^+^CD27^-^ B cells (**Figure 1H** and **Supplementary Figure 2H**). Interestingly, a heterogeneous pool of presumably short-lived effector (SLEf) B cells, negative for IgD and CD27, was transiently decreased during early convalescence (**Figure 1H** and **Supplementary Figure 2H**). Taken together, these data indicate that convalescence is paralleled by decrease of a pool of cytotoxic T cells likely containing cells responsive to SARS-CoV-2, and a gradual increase of naïve B cell counts.

### Spike-Specific B Cells Remain in Circulation

**Figure 2:**
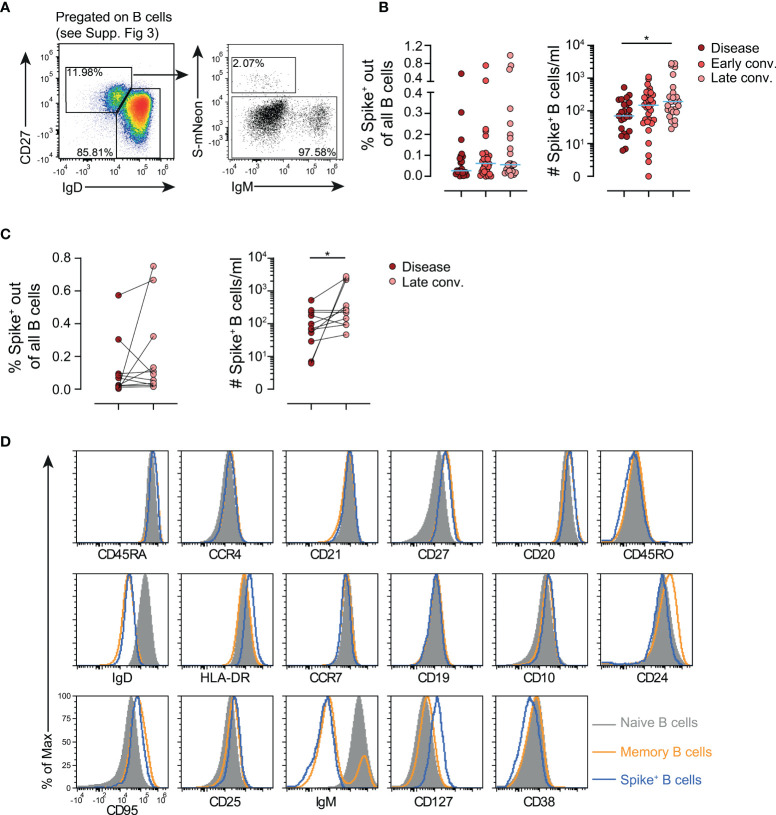
Deep profiling of circulating Spike- specific B cells by flow cytometry in patients. **(A)** Gating strategy for identification of Spike- specific B cells. Spike^+^ B cell gate was set based on the background level binding of CD3^+^ T cells. **(B)** Frequencies (left) and absolute numbers (right) of Spike- specific B cells in patients with disease or in early and late convalescence. Each dot represents one biologically individual sample taken from one patient. *n*(disease)=24; *n*(Early conv.)=32; *n*(Late conv.)=25. **(C)** Longitudinal analysis of frequencies (left) and absolute numbers (right) of Spike- specific B cells in patients 3-7 months after the disease. Each connected dot represent one individual patient (*n*=11). **(D)** Phenotype comparison of endogenous naïve and memory B cells and Spike- specific B cells. Shown is one representative sample from a patient in late convalescence. Statistics: Kruskal-Wallis multiple comparison test with Dunn’s *post hoc* correction. *p<0.05.

The presence of Spike-specific B cells following COVID-19 vaccination has already been demonstrated by several groups including ours (4, 11). However, there is a considerable lack of knowledge regarding the exact phenotype and the duration of persistence of these cells in SARS-CoV-2-infected patients. We therefore analyzed the Spike^+^ B cell compartment in the present cohort (**Figure 2A** and **Supplementary Figure 3**). As reported before, Spike- specific isotype-switched B cells already form during the disease phase (20), however they did not decline in frequencies nor numbers during early and late convalescence (**Figure 2B**). Also in the paired analysis of samples collected during the early, as well as late convalescent phase we also observed a moderate increase in absolute cell counts of Spike-specific B cells (**Figure 2C**) further indicating persistence of immunological memory. Spike^+^ isotype switched B cells actually showed a phenotype closely resembling that of endogenous memory B cells and only differ in increased CD127 expression (**Figure 2D**). These data indicate that Spike^+^ B cells are retained in numbers and frequencies in the circulation for at least 7 months, the longest observation period late convalescent patients were followed in the present study.

### Humoral anti-SARS-CoV-2 Responses Are Declining in Convalescing Patients

**Figure 3:**
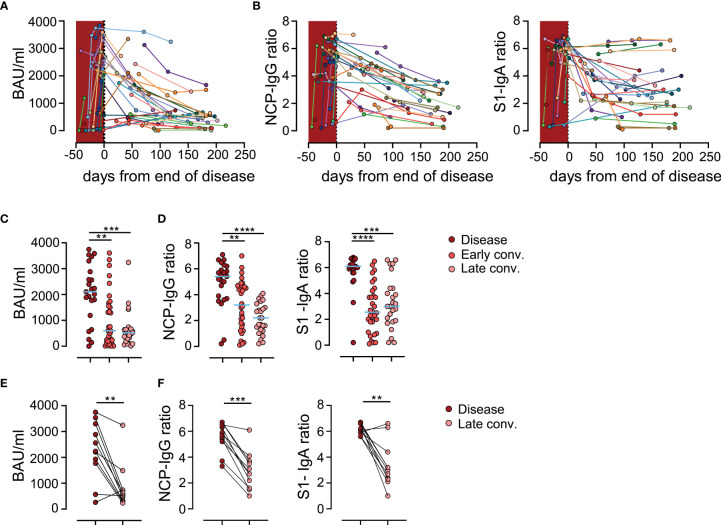
Anti-SARS-CoV-2 antibodies are declining in sera of convalescing patients. **(A)** Anti-Spike IgG levels in patients over time. Each individually colored dot and connecting line represent one patient (*n*=39 patients). Crimson shaded area represents the time during the disease. **(B)** Ratio of anti-nucleocapsid protein (NCP) IgG antibodies (left) and anti-Spike IgA (right) antibodies in patients over time. **(C)** Anti-Spike IgG levels in patients with disease, in early (early conv.) and late convalescence (late conv.). Each dot represents one biologically individual sample taken from one patient. *N* (disease)=23; *n* (early conv.)=32; *n* (late conv.)=25. **(D)** Ratio of anti-NCP IgG (left) and anti-Spike IgA (right) levels in patients. **(E)** Paired analysis of anti-Spike IgG levels in patients 3-7 months after the disease. Each connected dot represent one individual patient (*n*=11). **(F)** Paired analysis of ratios of anti-NCP IgG (left) and anti-Spike IgA (right) levels in patients 3-7 months after the disease. Statistics: **(C, D)** Kruskal-Wallis multiple comparison test with Dunn’s *post hoc* correction. **(E, F)** Two-tailed Wilcoxon matched-pairs signed rank test. BAU, Binding Antibody Units; **p<0.01; ***p<0.001; ****p<0.0001.

Longitudinal analysis of 39 patients revealed a decline in serum anti-Spike IgG levels over time in the majority of patients (**Figure 3A**). Similar declining trends were also observed for IgG antibodies recognizing the Nucleocapsid protein (NPC) as well as IgA antibodies recognizing the Spike protein (**Figure 3B**). In line with earlier reports (4, 21), we observed a significant reduction of anti-Spike and anti-NCP antibody levels during early and late convalescence (**Figures 3C, D**). To confirm the apparent reduction in humoral response during late convalescence, we performed an intra-patient paired analysis of antibody levels in patient samples collected during active disease with samples taken between 3 and 7 months post COVID-19 (**Figures 3E, F**). Data from this analysis were in line with results from our bulk analysis and revealed a steep decline of humoral responses in late convalescence samples. Taken together, these data confirm previous reports and point towards a substantial decline of antibody responses to SARS-CoV-2 with time (4, 6, 20, 22, 23).

### Sera From SARS-CoV-2 Wuhan Convalescents Show Waning Neutralization Capacity Against Alpha and Delta Variants of Concern

**Figure 4:**
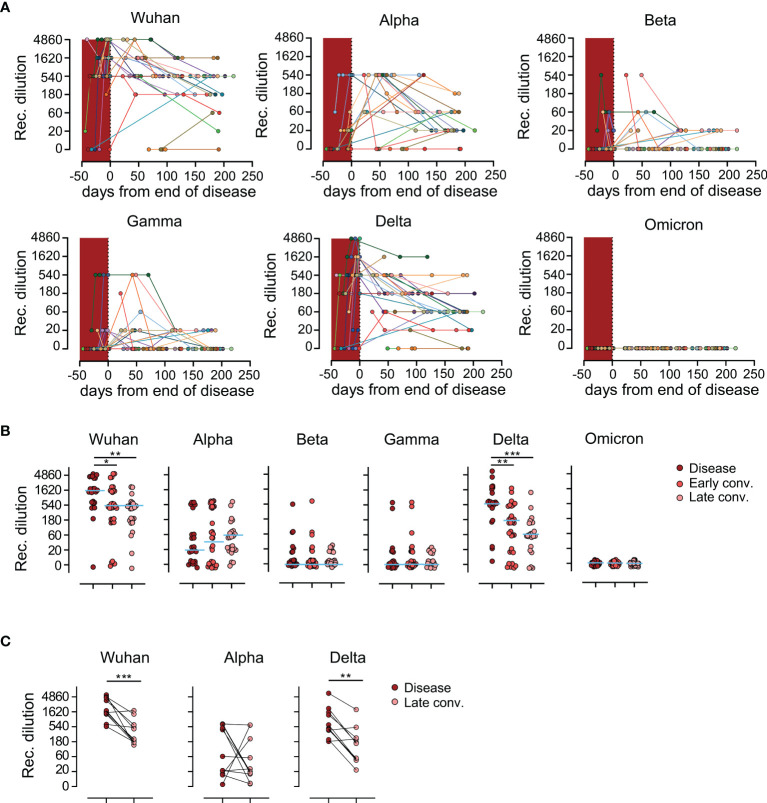
Waning of humoral protection against Wuhan strain and variants of concern. **(A)** Reciprocal titers of neutralizing antibodies against Wuhan strain, Alpha (B.1.1.7), Beta (B.1.351), Gamma (B.1.1.28.1), Delta (B.1.617.2) and Omicron (B.1.1.529) SARS-CoV-2-S variants over time, measured using the sVNT. Each individually colored dot and connecting line represent one patient (*n*=39 patients). Crimson shaded area represents time during the disease. **(B)** Reciprocal dilution titers of antibodies against Wuhan strain, Alpha, Beta, Gamma, Delta and Omicron variants. Each dot represents one biologically individual sample taken from one patient. *N* (disease)=23; *n* (early conv.)=32; *n* (late conv.)=25. **(C)** Longitudinal analysis of neutralizing antibodies titers in patients 3-7 months after the disease. Each connected dot represent one individual patient (*n*=11). **(B, C)** for better visualization of identical titer values, data were randomly and proportionally adjusted closely around the precise titer results. Statistics: **(B)** Kruskal-Wallis multiple comparison test with Dunn’s *post hoc* correction. **(C)** Two-tailed Wilcoxon matched-pairs signed rank test. *p<0.05; **p<0.01; ***p<0.001.

To determine the level of protection conferred by antibodies induced by infection with the original Wuhan strain of SARS-CoV-2 in convalescing patients to currently known VoCs on a functional level, we performed surrogate virus neutralization tests (sVNT) against the Wuhan strain as well as the Alpha, Beta, Gamma, Delta and Omicron VoC (10, 11). Applying the sVNT assay we observed strong differences in neutralization activity of patient’s sera against the original Wuhan strain and the five VoCs. While neutralizing antibodies against Wuhan, Alpha and Delta were detected in 37/39 (94.9%), 34/39 (87.2%), and 36/39 (92.3%) patients respectively, neutralization activity against Beta was detected in only 22/39 (56.4%), against Gamma in 18/39 (46.2%) and against Omicron in 0/39 (0%) patients across measured samples (**Figure 4A**). We then compared the neutralization capacity of patient’s sera against the different VoCs, in regard to disease and convalescence stages. This analysis revealed that sera showed the highest efficacy against the original Wuhan strain during the disease and both convalescence phases (**Figure 4B**). Overall, the convalescent sera exhibited very low to no neutralization capacity against the Beta, Gamma and Omicron VoCs at any time point tested, while the same sera possessed considerable neutralization efficacy against the Alpha and Delta variants. Interestingly, neutralization of Alpha was diminished compared to the Delta strain (**Figure 4B**). Moreover, while the neutralization efficacy against both the original Wuhan strain and the Delta VoC was highest in the disease phase and decreased over time, sera collected from individuals during the early or late convalescent phase had showed a tendency of increased neutralizing capacities against the Alpha VoC compared to sera taken during the disease phase (**Figure 4B**). These observations were further validated in the paired analysis (**Figure 4C**). These data indicate that antibodies produced during active disease are already able to neutralize the Wuhan strain and the Delta variant, while neutralization of the Alpha variant requires antibody affinity maturation.

### Total Anti-S1- IgG Levels Strongly Correlate With Neutralization Capacity

**Figure 5:**
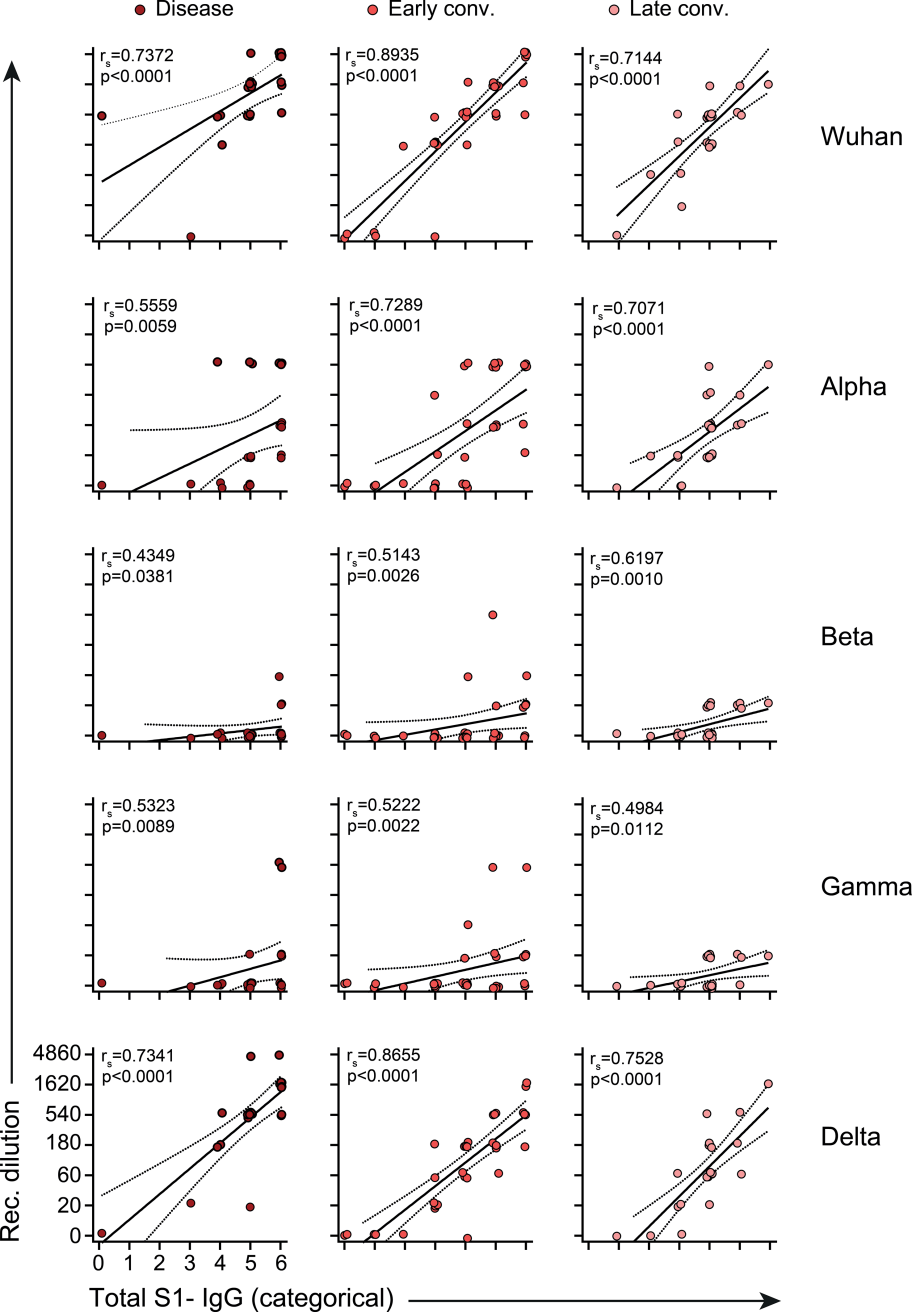
Correlate of total anti-Spike S1-IgG and neutralization antibody titers. Lines represent linear regression and dotted lines 95% confidence interval. Each dot represents one biologically individual sample taken from one patient. *N* (disease)=23; *n* (early conv.)=32; *n* (late conv.)=25. Absolute values of Spike-IgG (in RU/ml) were transformed into 7 categorical variables as described in **Supplementary Table 3**. For better visualization of identical titer values, data were randomly and proportionally adjusted closely around the precise titer results. Statistics: nonparametric Spearman correlation. r_s_= Spearman’s correlation coefficient.

While not being the sole factor, high antibody titers are often associated with functional protection. To test whether this also holds true for SARS-CoV-2 infection, we correlated categorically distributed levels of anti-Spike IgG with their neutralization capacity (**Figure 5**). For meaningful comparison we converted the absolute linear values of anti-S1 IgG to categorical values also applying 3-fold increases as done with the sVNT assays (**Supplementary Table 3**). This analysis revealed strong correlation of anti-S1 levels with sVNT titers against Wuhan, Alpha and Delta across all time points analyzed. In addition, correlation in the case of Wuhan and Delta did not prominently differ between the disease and convalescent phases (**Figure 5**). However in case of Alpha we observed a significant increase in correlation from disease to early and late convalescence (**Figure 5**), further indicating that affinity maturation is required for efficient neutralization of Alpha.

## Discussion

Here we analyzed humoral immune responses during active disease as well as in early and late convalescence of patients infected early during the pandemic with the Wuhan strain of SARS-CoV-2. We provide evidence that all these patients developed Spike-specific memory B cells that, on average, did not drop in frequency during the 7 months observation period. However at the same time, a considerable drop of neutralizing antibody titers against the Wuhan strain and the Alpha and Delta VoC was observed, while hardly any of the patients developed sizable neutralizing titers again the Beta and Gamma and none against the Omicron variants during disease or convalescence. The latter finding is of particular interest as it indicates that convalescent patients might be hardly protected from reinfections with the, Omicron variant this currently dominating in many countries. This finding is also reflected by observations made in vaccinees that also developed considerable lower neutralizing titers against Beta, Gamma and particular Omicron compared to Alpha, Delta or the Wuhan stain (12, 24, 25). Our results are also in line with data reported in publications indicating that COVID-19 convalescent individuals possess little if any serum antibodies able for neutralizing the Omicron variant (24, 26)

It seems likely that the B cell-mediated protection of infection is severely affected due to the high mutational load in the RBD domain of Omicron (9).

Unfortunately, even at this stage of the pandemic it is still difficult to exactly ascertain to what extent the different arms of the immune response contribute to the prevention of severe clinical manifestation of SARS-CoV-2 re-infection. Aside from antibodies, the three main sentinels providing SARS-CoV-2 immunity are NK cells, as well as Spike-specific T and B cells. A quantitative increase of NK cells in late convalescence and their switching towards a terminal phenotype observed in our cohort, indicate an important role of these cells in controlling SARS-CoV-2 infections. Several studies discuss different functional roles of terminally differentiated CD56^-^CD16^+^ NK cells, mostly associating them with poor cytotoxicity and antigen experience (14, 15, 27).

Our deep profiling of immune cells allowed us to quantify many B cell subsets, whose distinct proposed functions were reviewed in detail by others (28, 29) but we observed no major long-lasting effects on cell counts or frequencies of the various B cell subsets analyzed. However, we found numbers and frequencies of bulk antigen-experienced CD8^+^ T cell subsets to be transiently increased during convalescence. These findings are indicative of a normal contraction of the CD8^+^ T cell pool after the infection had been cleared. However, drawing definite conclusions about lymphocyte kinetics in this study is challenging as our study lacks healthy controls due to the difficulty in recruiting non-vaccinated age- and sex-matched individuals. Still, recent data suggests that the majority of patients do undergo restoration of homeostatic immunity during convalescence (30) and the values of major lymphocyte populations reported in this study also fall into the normal range for previously published healthy control cohorts (16, 31). In the present study we used cryopreserved PBMCs and therefore decided not to assess frequencies of virus-specific T cells. Re-stimulation of frozen PBMCs frequently results in IFN-gamma production by T cells even in the absence of specific peptides, making it difficult to determine rare events in already lymphopenic patients (32). This is presumably due to molecules released from damaged cells. Re-stimulation of previously frozen PBMCs with virus-specific peptides thus only allows rather limited conclusions about frequencies of T cells specific for SARS-CoV-2. Along these lines, CD8^+^ effector/memory type 1 cells (CD38^+^HLA-DR^+^) have been shown to contain Spike-responsive T cells (18, 19) and our results also demonstrate a strong decrease of this cell pool following late convalescence, implying the contraction of Spike-specific T cells from circulation. Similar kinetics for Spike-reactive T cells have been described in recent reports (33–35).

Spike-specific, isotype-switched B cells have been described in the blood of vaccinees (11) and of convalescent patients (4), but their exact phenotype and relevance in protecting from severe disease manifestation remain largely elusive. In contrast to serum antibody levels which decrease with time, we found even in late convalescence persisting frequencies or even increasing numbers of Spike-specific B cells. Our data indicate that these cells bear the phenotype of memory B cells and thus corroborate the results of a recent study (23). While the long-term presence of these cells is likely good news, it remains to be determined to what extent Spike- specific B cells ameliorate COVID-19. Since only antibodies on mucosal surfaces can prevent infection, it seems unlikely that memory B cells play a role at the very early step of the infection process.

While the level of protection conveyed by cellular immunity alone is difficult to ascertain, it seem clear that high levels of neutralizing antibodies are a potent indicator of protection against severe course of COVID-19 (36). Interestingly, the kinetics of neutralization efficacy against the Wuhan strain closely resembled those against the Delta strain, but were different for the Alpha strain. While during late convalescence, 95%, 87% and 92% of our patients possessed antibodies capable of neutralizing the Wuhan strain, the Alpha and Delta VoC respectively, the kinetics of the response differed between the 3 strains. During the disease phase 8/24 (33.3%) patients did not develop antibodies, able to neutralize the Alpha variant which was only the case in 1/24 (4%) patients for the Wuhan and Delta variants.

Taken together, our data corroborate previous findings by other groups reporting anti-virus antibody decay in convalescing patients, indicating the functional decrease of protection. While the presence of neutralizing antibodies against Wuhan, Alpha and Delta variants is encouraging, a complete lack of neutralization against the Omicron variant observed even during peak response indicates viral escape and subsequent reduced immune protection against this variant as well as Beta and Gamma. Our findings corroborate reports from others of reduced efficacy of current vaccines and convalescent sera against the aforementioned variants (6, 25, 37–39). Furthermore, bona fide memory Spike- specific B cells seem to remain in circulation for long periods of time post COVID-19, implying a certain layer of defense against severe disease, even in patients with low antibody titers. Vaccination provides good protection against severe forms of COVID-19 and more studies are urgently needed to evaluate the change and duration of immune responses in convalescing people who got vaccinated.

## Data Availability Statement

The raw data supporting the conclusions of this article will be made available by the authors, without undue reservation.

## Ethics Statement

The studies involving human participants were reviewed and approved by Institutional review board at Hannover Medical School (#9001_BO_K2020). The patients/participants provided their written informed consent to participate in this study.

## Author Contributions

RF designed the study. CS-F, RS, LB, CK, and MC collected clinical data. IO, SH, CR, SW, MF, IR, RG, MS, AC, and BB performed experiments. GB, GH, and TK synthesized reagents. IO, AC, and SH analyzed experimental data, IO, CS-F, BB, GMNB, and RF interpreted the data. IO and CS-F wrote the manuscript. RF reviewed and edited the manuscript. All authors read and approved the final version of the manuscript.

## Funding

The study was supported by DZIF TTU 01.938 (grant 80018019238 to RF and GB), by DZL (grant 82DZL002B1 to RF), by DFG EXC 2155 “RESIST” (Project ID39087428 to RF), SFB 900/3 (Projects B1, 158989968 to RF and project B8 to CK) and FO334/7-1 to RF, by the State of Lower Saxony (14-76103-184 CORONA-11/20 to RF and TK) by the BMBF (NaFoUniMedCovid19 FKZ: 01KX2021; Project B-FAST to RF), and by the European Regional Development Fund (Defeat Corona, ZW7-8515131 and ZW7-85151373 to GB). The funding sources of this study did not play any role in the study design, the collection, analysis and interpretation of the data, in the writing of the manuscript and in the decision to submit the paper for publication. The authors have not been paid by a pharmaceutical company or other agency to write this article. The corresponding author confirms that he had full access to all data in the study and had final responsibility for the decision to submit for publication.

## Conflict of Interest

The authors declare that the research was conducted in the absence of any commercial or financial relationships that could be construed as a potential conflict of interest.

## Publisher’s Note

All claims expressed in this article are solely those of the authors and do not necessarily represent those of their affiliated organizations, or those of the publisher, the editors and the reviewers. Any product that may be evaluated in this article, or claim that may be made by its manufacturer, is not guaranteed or endorsed by the publisher.
