# Supplementary material for: Longitudinal Tracking of Immune Responses in COVID-19 Convalescents Reveals Absence of Neutralization Activity Against Omicron and Staggered Impairment to Other SARS-CoV-2 Variants of Concern
Source: Front Immunol. 2022 Mar 14;13:863039. doi: 10.3389/fimmu.2022.863039 (PMC8964088; doi:10.3389/fimmu.2022.863039)
Supplement: Supplementary file 1 [file DataSheet_1.docx]

**Supplementary Material**

| **ID** | **Disease** | **Early convalescence** | **Late convalescence** |
| --- | --- | --- | --- |
| 002 | 2 | 2 | 1 |
| 003 |  | 1 | 1 |
| 004 |  | 1 | 1 |
| 005 | 1 | 1 |  |
| 007 |  | 1 | 1 |
| 008 |  | 1 | 1 |
| 009 |  | 1 | 1 |
| 010 |  | 2 | 1 |
| 011 |  | 1 | 2 |
| 012 | 2 |  | 1 |
| 013 |  | 1 |  |
| 014 | 3 | 1 |  |
| 015 |  | 2 | 1 |
| 016 | 1 |  | 2 |
| 017 |  | 1 |  |
| 018 |  | 2 |  |
| 019 | 1 | 2 |  |
| 020 |  | 1 | 1 |
| 021 |  | 1 | 2 |
| 022 | 1 |  |  |
| 023 | 2 |  |  |
| 024 |  | 1 | 1 |
| 025 | 2 |  |  |
| 026 |  | 1 | 1 |
| 027 | 3 | 1 | 1 |
| 028 | 1 |  |  |
| 029 | 2 | 1 | 1 |
| 030 |  | 1 | 2 |
| 032 | 1 |  | 1 |
| 033 | 1 |  | 2 |
| 034 | 2 | 2 | 1 |
| 036 |  |  | 2 |
| 037 |  | 1 |  |
| 038 | 3 |  |  |
| 039 | 2 |  |  |
| 040 | 2 |  |  |
| 041 | 3 | 2 |  |
| 042 | 3 | 1 | 1 |
| 043 |  | 1 |  |
| 044 | 2 | 1 |  |
| 045 |  | 2 | 1 |
| 046 | 2 | 2 |  |
| 047 |  | 1 | 1 |
| 048 |  | 1 |  |
| 049 | 3 |  |  |
| 050 | 3 |  |  |

**Supplementary Table 1.** Overview of patient samples. Disease was defined as any sample where active COVID-19 symptoms were present. Early and late convalescence were defined as samples taken up to 4 months (early) and more than 4 months (late) after the clearance of disease.

|  | **Antigen** | | **Fluorophore** | **RRID** | **Clone** | **Order No.** | **Company** | **Dilution** |
| --- | --- | --- | --- | --- | --- | --- | --- | --- |
| 1 | CD45RA | | BUV395 | AB_2740037 | HI100 | 740298 | BD | 1:100 |
| 2 | CD16 | | BUV496 | AB_2870224 | 3G8 | 612944 | BD | 1:100 |
| 3 | CD4 | | BUV563 | AB_2870854 | RPA-T4 | 741353 | BD | 1:100 |
| 4 | CCR4 | | BUV615 | AB_2870269 | 1G1 | 613000 | BD | 1:20 |
| 5 | CD21 | | BUV661 | AB_2874389 | 1048 | 750187 | BD | 1:20 |
| 6 | CD56 | | BUV737 | AB_2813880 | NCAM16.2 | 612766 | BD | 1:20 |
| 7 | CD27 | | BUV805 | AB_2873108 | L128 | 748704 | BD | 1:20 |
| 8 | CD20 | | BV 421 | AB_10965543 | 2H7 | 302330 | Biolegend | 1:20 |
| 9 | CD45RO | | Pacific Blue | AB_493659 | UCHL1 | 304216 | Biolegend | 1:100 |
| 10 | IgD | | BV 480 | AB_2739536 | IA6-2 | 566138 | BD | 1:20 |
| 11 | CD62L | | VioGreen | AB_2726192 | 145/15 | 130-113-623 | Miltenyi | 1:50 |
| 12 | HLA-DR | | BV 570 | AB_2650882 | L243 | 307638 | Biolegend | 1:20 |
| 13 | TCRVg9 | | BV 605 | AB_2741946 | B3 | 744036 | BD | 1:20 |
| 14 | CCR6 | | BV 711 | AB_2629608 | G034E3 | 353436 | Biolegend | 1:20 |
| 15 | CXCR5 | | BV 750 | AB_2871862 | RF8B2 | 747111 | BD | 1:20 |
| 16 | CCR7 | | BV 785 | AB_2563630 | G043H7 | 353230 | Biolegend | 1:20 |
| 17 | Spike-mNeonGreen | | mNeonGreen | N/A | N/A | N/A | N/A | 5µg/sample |
| 18 | CD3 | | AF532 | AB_11218675 | UCHT1 | 58-0038-42 | Invitrogen | 1:20 |
| 19 | CD8 | | SparkBlue550 | AB_2819983 | SK1 | 344760 | Biolegend | 1:100 |
| 20 | CD19 | | PerCP | AB_893272 | HIB19 | 302228 | Biolegend | 1:20 |
| 21 | CD14 | | BB700 | AB_2739737 | MφP9 | 566465 | BD | 1:100 |
| 22 | CD10 | | PE | AB_314915 | HI10a | 312204 | Biolegend | 1:20 |
| 23 | CD24 | | PE/Dazzle 594 | AB_2566349 | ML5 | 311134 | Biolegend | 1:20 |
| 24 | CD138 | | PE-eF610 | AB_2815308 | MI15 | 61-1388-42 | Invitrogen | 1:20 |
| 25 | CD95 | | PE-Cy5 | AB_314548 | DX2 | 305610 | Biolegend | 1:20 |
| 26 | CD25 | | PE-Cy7 | AB_314282 | BC96 | 302612 | Biolegend | 1:20 |
| 27 | TCRgd | | APC | AB_2733463 | 11F2 | 130-113-500 | Miltenyi | 1:50 |
| 28 | IgM | | Af647 | AB_2566613 | MHM-88 | 314536 | Biolegend | 1:20 |
| 29 | CD127 | | AF700 | AB_2566200 | A019D5 | 351344 | Biolegend | 1:20 |
| 30 | viability | | Zombie NIR |  | N/A | 423106 | Biolegend | 1:400 |
| 31 | CD38 | | APCe780 | AB_11218674 | HIT2 | 47-0389-42 | Invitrogen | 1:20 |
|  |  | |  |  |  |  |  |  |
|  | | **Supplementary Table 2**: Antibodies and reagents used for flow cytometry | | | | | |  |

| Value range (RU/ml) | Category |
| --- | --- |
| ≤3 | 0 |
| <3 **X** ≤9 | 1 |
| <9 **X** ≤27 | 2 |
| <27 **X** ≤81 | 3 |
| <81 **X** ≤243 | 4 |
| <243 **X** ≤729 | 5 |
| <729 | 6 |

**Supplementary Table 3: Transformation of continuous to categorical variables**

**Supplementary Figure 1**: **Gating strategies of flow cytometry analysis**.

**Supplementary Figure 2**: **Frequencies of leukocyte subpopulations in convalescing patients.** Cell frequencies in patients with disease, early and late convalescence **a**) Frequencies of various leukocyte populations **b**) Frequencies of major T cells subsets **c**) Frequencies of NK cell subsets. **d**) Frequencies of monocyte subsets. **e**) Frequencies of memory populations of conventional CD4^+^ T cells. **f**) Frequencies of memory subsets of conventional CD8^+^ T cells. **g**) Frequencies of CD8^+^CD38^+^HLA-DR^+^ effector memory subpopulation. **h**) Frequencies of B cell subsets. Each dot represents one biologically individual sample taken from one patient. *n*(disease)= 24; *n*(Early conv.)= 32; *n*(Late conv.)= 25. Statistics: Kruskal-Wallis multiple comparison test with Dunn’s post hoc correction. *p<0.05; **p<0.01; ***p<0.001; ****p<0.0001

**Supplementary Figure 3:** **Pre-gating strategy for analysis of spike- specific B cells.**

**Supplementary Material and Methods**

*Cohort Charactheristics*

Median time of disease was 21 days (range 14-55 days) and 50% of all patients required non-invasive or mechanical ventilation (WHO ordinal scale ≥5). Patient samples were analyzed by three different approaches: 1) bulk analysis of all samples available meeting the definition for either active disease (n=23), early convalescence (early conv., n=32) or late convalescence (late conv., n=25); 2) paired analysis of patients with availability of a disease and late convalescence sample (n=11) and 3) longitudinally, including all patients with 2 or more consecutive samples (n=39). ‘Disease sample’ was defined as any sample collected while active COVID-19 symptoms were present. Convalescence samples were defined as “early” when taken up to 4 months and “late” when collected more than 4 months after clearance of the disease. In cases of multiple samples from the same patient, of the same category (disease, early conv. or late conv.) the latest sample was used in the analysis (Supplementary Table 1). Patient samples without complete blood count or Anti-Spike IgG levels <10 RU/ml during active disease in case of missing follow-up samples were excluded, leading to a total of 46 study participants with a total of 120 samples eligible for study analysis (Supplementary Table 1).
